# Supplementary material for: Shifting Paradigm from Gene Expressions to Pathways Reveals Physiological Mechanisms in Blood Pressure Control in Causation
Source: Int J Mol Sci. 2023 Jan 9;24(2):1262. doi: 10.3390/ijms24021262 (PMC9863686; doi:10.3390/ijms24021262)
Supplement: Supplementary file 1 [file ijms-24-01262-s001.zip › ijms-2062741-supplementary.pdf]

Shifting paradigm from gene expressions to pathway reveals causes of physiological mechanisms in blood pressure control by quantitative trait loci

Alan Y. Deng, Annie Menard and David W. Deng

Research Centre, CRCHUM (Centre hospitalier de l'Université de Montréal), Department of Medicine, Université de Montréal, Montréal, Québec, Canada

Table Supplementary S1 (Table S1): Rat QTLs and genes residing in the chromosome intervals harboring them that correspond to human GWAS genes functionally captured by congenic knock in genetics.

Rat QTL	Rat Gene	Rat Chr spans	Human GWAS gene	Human CHR
C1QTL1	***Fndc1***	48905631..48987877	***FNDC1***	6
C1QTL2	*Pepd*	91925878.. 92416063	*PEPD*	19
	Mybpc2	101572091..101595585	*MYBPC2*	19
	Rras	102077775..102081673	*RRAS*	19
	Snrnp70	102431122..102468602	*SNRNP70*	19
	Kcnj11	103186859..103190535	*KCNJ11*	11
	Atp10a	117144423..117311749	*ATP10A*	15
	Qpctl	81314398..81323907	*QPCTL*	19
	Gipr	81329939..81340229	*GIPR*	19
	Apoe	81878372..81882298	*APOE*	19
	B9d2	83735237..83741209	*B9D2*	19
	Akt2	85617612..85667423	*AKT2*	19
	Fbxo17	88084918..88100373	*FBXO17*	19
	Sars2	88104901..88116638	*SARS2*	19
	Tshz3	93825005..93896330	*TSHZ3*	19
	Ccne1	95575136..95584387	*CCNE1*	19
C1QTL3	*Mef2a*	129273893..129407071	*MEF2A*	15
	Mctp2	134362777..134596330	*LOC400456*	15
	Chd2	135782462..135897354	*CHD2*	15
	Akap13	138007112..138312524	*AKAP13*	15
	Klhl25	138322710..138355032	*KLHL25*	15
	Idh2-Sema4b	142830040.. 143153628	*IDH2[21861]-SEMA4B*	15
	Furin-Fes	14312727..143136513	*FURIN-FES*	15
	Zscan2	143630849..143646301	*ZSCAN2*	15
	Wdr73	143663956..143672463	*WDR73*	15
	Nmb	143673434..143676833	*NMB*	15
	Pde8a	143980312..144102752	*PDE8A*	15
	Tm6sf1	144601683..144647599	*TM6SF1*	15
	Adamtsl3	145170122..145490045	*ADAMTSL3*	15
	Abhd17c	147177240..147218200	*ABHD17C*	15
	Nox4	157246099..157285107	*NOX4*	15
	Gab2	168423264..168486051	*GAB2*	11
	Usp35	168490390..168514120	*USP35*	11
	Clns1a	168883031..168903326	*CLNS1A*	11
	Thap12	169866610..169882330	*C11orf30*	11
	Pold3	171284462..171323431	*POLD3*	11
	Fam168a	171925433..172078008	*FAM168A*	11
	Relt	172081904..172099491	***RELT (C1QTL5RELT)***	11
	Arhgef17	172105535..172164079	*ARHGEF17*	11
	P2ry6	172169438..172204831	*P2RY6*	11
	Clpb	172929476..173055807	*CLPB*	11

	Phox2a	173083448..173087966	*PHOX2A*	11
	Cckbr	177269044..177279480	*CCKBR*	11
	Lmo1	180718642..180753931	*LMO1*	11
	St5	181125790..181275226	*ST5*	11
	Arntl	185007527..185105413	*ARNTL*	11
	Adm	182433109..182435280	*ADM*	11
	Nucb2	192078545..192115045	*NUCB2*	11
	Plekha7	192397589..192463974	*PLEKHA7*	11
	Sox6	192644288..193155831	*SOX6*	11
	Umod	196127939..196141822	***UMOD* (*C1QTL6UMOD*)**	16
	Tnrc6a	200079394..200206235	*TNRC6A*	16
	Fbxl19	206218078..206238886	*FBXL19*	16
	Bag3	206989244..207013177	***BAG3* (*C1QTL7BAG3*)**	10
	Fgfr2	207626732..207731841	*FGFR2*	10
	Htra1	208532013..208581538	*HTRA1*	10
	Ctbp2	211993864..212023075	*CTBP2*	10
	Ppp2r2d	218132463..218166450	*PPP2R2D*	10
	Bnip3	218174697..218191883	*BNIP3*	10
	Inpp5a	218656022..218844837	*INPP5A*	10
	Cracr2b	220920617..220926199	*CRACR2B*	11
	H19	222639223..222641899	*H19*	11
	Ano1	224612597..224760421	*ANO1*	11
	Sipa1	227941623..227953709	*SIPA1*	11
	Ehbp1l1	227997200..228017390	*EHBP1L1*	11
	Cox8a	229457144..229459465	*COX8A*	11
	Lrrc10b	233448290..233449518	*LRRC10B*	11
	Cbwd1	250092601..250135008	***CBWD1* (*C1QTL8CDK*)**	9
	Dock8	250200507..250417771	***DOCK8***	9
	Kank1	250464788..250659569	***KANK1***	9
	Glis3	253622178..254039761	*GLIS3*	9
	Pten-Rnls	258651829..258717009-259052388..259324901	*PTEN-RNLS*	10
	Hhex	263583988..263589575	*HHEX*	10
	Plce1	264652842..264956664	*PLCE1*	10
C18QTL2	*Tmco6*	29282952..29289547	*TMCO6*	5
	Ndufa2	29289478..29291567	*NDUFA2*	5
	Ik	29291708..29306512	*IK*	5
	Wdr55	29309024..29312537	*WDR55*	5
	Dnd1	29312395..29315019	*DND1*	5
	Hars	29315352..29332458	*HARS*	5
	Hars2	29332574..29341926	*HARS2*	5
	Zmat2	29343457..29348092	*ZMAT2*	5
	Pcdha1	29648344..29924443	*PCDHA1*	5
	Pcdha2	29657322..29924443	*PCDHA2*	5
	Pcdha3	29663495..29924443	*PCDHA3*	5
	Pcdha4	29670058..29924443	*PCDHA4*	5
	Pcdha5	29677518..29924444	*PCDHA5*	5
	Pcdha6	29684456..29924443	*PCDHA6*	5

	Pcdha7	29690611..29924443	*PCDHA7*	5
	Prr16	45166501..45372242	*PRR16*	5
	Ftmt-Srfbp1	46510407..46690474	*FTMT-SRFBP1*	5
	Prdm6	47648048..47750565	*PRDM6*	5
	Csnk1g3	48043786..48126393	*CSNK1G3*	5
	Slc12a2	52104434..52172638	*SLC12A2*	5
	Fbn2	52284482..52487739	*FBN2*	5
C18QTL3	*Fbxo38*	57053464..57100790	*FBXO38*	5
	Spink7	57142773..57146245	*SPINK7*	5
	Piezo2	57576278..57949635	*PIEZO2*	18
	Wdr7	58326473..58612197	*WDR7*	18
	Nedd4l	59595643..59921504	*NEDD4L*	18
C18QTL4	*Slc14a2*	74185426..74617691	*SLC14A2*	18
	Setbp1	74772163..74960529	*SETBP1*	18
	Tshz1	79910848..79956064	*TSHZ1*	18

Fig. 1 of the text defines the chromosome regions containing QTLs and the gene residing in the intervals containing these QTLs. Human GWAS genes are from (Evangelou et al., 2018). Only the genes underlined were analyzed further in the current work, because they are functional candidate genes for the human QTLs. ***Arhgap12*,** Rho GTPase activating protein 12; ***Arhgef17*,** Rho guanine nucleotide exchange factor 17; ***Bag3*,** BAG cochaperone 3; ***Cbwd1*,** COBW domain containing 1; ***Dnd1*,** DND microRNA-mediated repression inhibitor 1; ***Dnmt3b*,** DNA methyltransferase 3 beta; ***Fndc1***, fibronectin type III domain containing 1; ***Kank1***, KN motif and ankyrin repeat domains 1; ***Dock8*,** dedicator of cytokinesis 8; ***Plce1*,** phospholipase C, epsilon 1; ***Procr*,** protein C receptor; ***Relt*,** RELT, TNF receptor; ***Rreb1*,** ras responsive element binding protein 1; ***Umod*,** uromodulin; ***Zscan2***, zinc finger and SCAN domain containing 2. CHR, Chromosome.

Table Supplementary S2 (Table S2): Mutation survey of genes in rat QTL-residing intervals that capture human GWAS genes by congenic knock in genetics

Rat QTL name	Rat/human GWAS Gene	Mutation detected Lew/ DSS	Change in amino acid (AA) Lew/DSS	RGD (AA) Lew/DSS	Human GWAS SNP
C1QTL1	***FNDC1***	C282T<br>C1155T<br>T1169C<br>C1174T<br>A1330G<br>G2526T<br>C2900G<br>C3198G<br>G3447A<br>C4121T<br>C4140G<br>A4158G<br>C4197T<br>T4437C<br>T4450C	No<br>No<br>**M390T**<br>**P392S**<br>**N444D**<br>No<br>**P967R**<br>No<br>No<br>**P1374L**<br>No<br>No<br>No<br>No<br>No	 <br>**M390T**<br>**P392S**<br>**N444D**<br><br>No<br><br><br>**P1475L**	Intergenic<br>rs449789
C1QTL2	*Thz3*	C2784T	No	No	
C1QTL3	*Mctp2*	C474T<br>T558C<br>G1017A<br>C2457A	No<br>No<br>No<br>No	No<br>No<br>No<br>No	
	Akap13	3'UTR	No	No	
	Idh2	3'UTR	No	No	
	Sema4b	A1203G<br>A1419G	No<br>No	No<br>No	
	Fes	C717T	No	No	
	Furin	C1066T	No	No	
	Zscan2	No	No	E155K unconfirmed	rs3825877 intergenic
	Pde8a	T1410C	No	No	
	Gab2	T152C	No	No	
	Clns1a	5'UTR<br>3'UTR	No<br>No	No<br>No	
	Prkrir	3'UTR	No	No	
	Fam168a	T552C	No	No	
	Relt	G214A<br>C522G	**G72S**<br>No	**G72S**<br>No	rs7115605 (intergenic)
	Arhgef17	No	No	G1016S unconfirmed	rs11827650 (intronic)
	P2ry6	3'UTR	No	No	
	P2ry2	5'UTR<br>T999C	No<br>No	No<br>No	

		3'UTR	No	No	
	Phox2a	T360C	No	No	
	Dennd2b (St5)	C2457T	No	No	
	Arntl	5'UTR	No	No	
	Nucb2	G150A<br>T1176C	No<br>No	No<br>No	
	Umod	C51T<br>G253A<br>C464T<br>C469G<br><br>C669A<br>T994C<br>T1422C	No<br>**V85M**<br>**S155F**<br>E157Q<br>(unconfirmed)<br>No<br>**I326V**<br>No	No<br>**V85M**<br>**S155F**<br>No<br><br>No<br>**I326V**<br>No	 rs13333226<br>(intronic)
	Tnrc6a	3'UTR	No	No	
	Fbxl19	3'UTR	No	No	
	Bag3	C957T<br>C994T<br>T1225C<br>A1256C<br>T1332C	No<br>**P332S**<br>**S409P**<br>**H419P**<br>No	**No**<br>**P332S**<br>**S409P**<br>**H419P**<br>No	rs72842207<br>(intronic)
	Fgfr2	3'UTR	No	No	
	Ctbp2	3'UTR	No	No	
	Ppp2r2d	A468G	No	No	
	Bnip3	3'UTR	No	No	
	Inpp5a	G994A	No	No	
	H19	No cDNA	No	No	
	Cbwd1	G242A	**S81N**	**S81N**	rs2992854<br>(intergenic)
	Dock8	G437A<br>T1797G<br>C2027T<br>G3675A<br>T3876C<br>G4296A<br>C4365T<br>T4413C	**R146K**<br>No<br>No<br>No<br>No<br>No<br>No<br>No	**R146K**<br>No<br>No<br>No<br>No<br>No<br>No<br>No	rs643058<br>(intergenic)<br><br>rs520015<br>(intronic)<br><br>rs604470<br>(intronic)
	Kank1	C210T<br>C567T<br>A1463G<br>T1616C<br>C1701T<br>T1983C<br>C2465G<br>G3126A<br>G3902G	No<br>No<br>No<br>**M539T**<br>No<br>No<br>**P822R**<br>No<br>**S1301N**	No<br>No<br>No<br>**M539T**<br>No<br>No<br>**P820A**<br>No<br>**S1299N**	 rs17369029<br>(3'UTR)<br><br>rs16923342<br>(intergenic)<br><br>rs60191654<br>(intergenic)
	Plce1	T2103C<br>C3522T	No<br>No	No<br>No	rs932764<br>(intronic)

		T3597C<br>C3675T<br>G3697A	No<br>No<br>Ala1233Thr (unconfirmed)	No<br>No<br>No	
C18QTL2	*Tmco6*	5'UTR	No	No	
	Ndufa2	G135A	No	No	
	Ik	T342C	No	No	
	Dnd1	C864T<br>G866A<br><br>G877A	No<br>289Stop (unconfirmed)<br>No	No<br>No<br><br>No	rs2530243 (intronic)<br><br>rs2563333 (synonymous)
	Hars2	C1431T	No	No	
	Pcdha1	Not sure		Not sure	
	Pcdha2	Not sure		Not sure	
	Pcdha3	Not sure		Not sure	
	Pcdha4	Not sure		Not sure	
	Pcdha5	Not sure		Not sure	
	Pcdha6	Not sure		Not sure	
	Pcdha7	Not sure		Not sure	
	Prr16	A84G	No	Not sure	
	Csnk1g3	T461C<br>C689T	No<br>No	No<br>No	
	Slc12a2	G462A<br>G2187A	No<br>No	No<br>No	
	Fbn2	T741C<br>A3710G<br>C3704T<br>C3986T<br>C4781G<br>C5882T<br>A8533G<br>T8534C	No<br>No<br>No<br>No<br>No<br>No<br>No<br>No	No<br>No<br>No<br>No<br>No<br>No<br>No<br>No	
C18QTL4	*Nedd4l*	A321T<br>T1995C<br>A2139G	No<br>No<br>No	No<br>No<br>No	

Gene locations are indicated on the map in Fig. 1. The position of a mutation enumerates from the ATG start codon of that gene. The amino acid position begins from the first methionine. ***Arhgap12*****,** Rho GTPase activating protein 12; ***Arhgef17*****,** Rho guanine nucleotide exchange factor 17; ***Bag3*****,** BAG cochaperone 3; ***Cbwd1*****,** COBW domain containing 1; ***Dnd1*****,** DND microRNA-mediated repression inhibitor 1; ***Dnmt3b*****,** DNA methyltransferase 3 beta; ***Fndc1***, fibronectin type III domain containing 1; ***Kank1***, KN motif and ankyrin repeat domains 1; ***Dock8*****,** dedicator of cytokinesis 8; ***Plce1*****,** phospholipase C, epsilon 1; ***Procr*****,** protein C receptor; ***Relt*****,** RELT, TNF receptor; ***Rreb1*****,** ras responsive element binding protein 1; ***Umod*****,** uromodulin; ***Zscan2***, zinc finger and SCAN domain containing 2. No Copy Number Variation (CNV) had been found for those genes from total genome sequencing of DSS and Lewis rats based on our current work and those of the rat genome database. RGD, public rat genome base.

Table Supplementary S3 (Table S3): Survey of non-coding GWAS SNP conservations/homology during primate evolution

More recent ← More ancient

**Human SNP/ Marked gene**	**Chimpanzee** (6.4 MYA)	**Bonobo** (6.4 MYA)	**Gorilla** (8.6 MYA)	**Orangutan** (15.2 MYA)	**Gibbons** (19.6 MYA)	**Old World monkeys** (28.9 MYA)	**New World monkeys** (43 MYA)
rs449789/*FNDC1* (intergenic)	homology	homology	homology	homology	homology	homology/ rhesus; No homology/ baboon	No homology
rs7115605/*RELT* (intergenic variant)	homology	homology	homology	homology	homology	homology	Homology/ Marmoset No homology/ Squirrel Monkey; SNP absent, and the surrounding sequence is not conserved.
rs13333226/*UMOD* (intron variant)	homology	homology	homology	homology	homology	homology	homology
rs72842207/*BAG3* (intron variant)	homology	homology	homology	homology	homology	homology	No homology; SNP absent, but the surrounding sequence is little conserved.
rs2992854/*CBWD1* (intergenic variant)	No homology; SNP absent, no hit on the right chr.	homology	homology	homology	homology	homology	homology
rs643058/*DOCK8* (intergenic variant)	No homology; SNP absent, no hit on the right chr.	No homology ;SNP absent, and sequence around is not conserved.	homology	homology	homology	homology	Homology/Ma rmoset No homology/ Squirrel Monkey; SNP absent, and the surrounding sequence is not conserved.
rs520015/*DOCK8* (intron variant)	No homology; SNP absent, no hit on the right chr.	homology	homology	homology	homology	homology	No homology; SNP absent, but the surrounding sequence is little conserved.
rs604470/*DOCK8* (intron variant)	homology	homology	homology	homology	homology	homology	homology
rs17369029/*KANK1* (3'UTR variant)	homology	No homology ; SNP absent, but surroundin g sequence	homology	homology	homology	homology	homology

		is little conserved.					
rs16923342/*KANK1* (intergenic variant)	homology	homology	homology	homology	homology	No homology; SNP absent, but the surrounding sequence is much conserved.	No homology; SNP absent, but the surrounding sequence is little conserved.
rs60191654/KANK1 (intergenic variant)	homology	No homology ; SNP absent, no hit on the right chr.	homology	homology	homology	homology	homology

Gene names are given in the legend for Table 1. Homology indicates that the SNP and/or surrounding sequences are conserved. Old World monkeys are represented by Rhesus macaque, baboon; New World monkeys are represented by marmoset, squirrel monkey. Searches were done at https://genome.ucsc.edu/cgi-bin/hgGateway. MYA, millions of years in divergence to humans (https://timetree.org/).

Table Supplementary S4 (Table S4): Amino acid alignment and missense mutations in codons for fibronectin type III domain containing 1 (FNDC1) in humans and rats.

```
Human FNDC1        MAPEAGATLRAPRRLSWAALLLLAALLPVASSAAASVDHPLKPRHVKLLSTKMGLKVTWD    60
DSS rat Fndc1      ----------------------------------------------------MGLKVTWD    8
                                                                       ********

Human FNDC1        PPKDATSRPVEHYNIAYGKSLKSLKYIKVNAETYSFLIEDVEPGVVYFVLLTAENHSGVS    120
DSS rat Fndc1      PPKDATSRPVEHYNIAYGKSLKSLKSIKVNAETHSFLIKDV-------------------    49
                   ************************* *******:****:**

Human FNDC1        RPVYRAESPPGGEWIEIDGFPIKGPGPFNETVTEKEVPNKPLRVRVRSSDDRLSVAWKAP    180
DSS rat Fndc1      ---------------------------------EKEVPNKPLRMRVRASDDRLSVAWKAP    76
                                                    **********:***:************

Human FNDC1        RLSGAKSPRRSRGFLLGYGESGRKMNYVPLTRDERTHEIKKLASESVYVVSLQSMNSQGR    240
DSS rat Fndc1      RLSGAKSPRRSRGFLLGYGESGRKMNYVPLTRDERSHEIKKLASESVYVVSLQSTNSQGQ    136
                   ***********************************:****************** ****:

Human FNDC1        SQPVYRAALTKRKISEEDELDVPDDISVRVMSSQSVLVSWVDPVLEKQKKVVASRQYTVR    300
DSS rat Fndc1      SQPVYRAALTKRKNAEEDELDVPEDISVRVMSSQSVLVAWVDPLVEKQKRVVASRQYTVR    196
                   ************* :********:**************:****::****:**********

Human FNDC1        YREKGELARWDYKQIANRRVLIENLIPDTVYEFAVRISQGERDGKWSTSVFQRTPESAPT    360
DSS rat Fndc1      YREKGESARWDYKQVSNRRALVDSLIPDTVYEFAVRISQGERDGKWSASVFQRTPESAPT    256
                   ****** *******::***.*::.***********************:************

Human FNDC1        TAPENLNVWPVNGKPTVVAASWDALPETEGKVKEYILSYAPALKPFGAKSLTYPGDTTSA    420
DSS rat Fndc1      TAPENLRVWPVNGKPTVVTVSWDALPESEGKVKEYILSYAPALKPFGAKSLTFSGHTTSA    316
                   ******.***********:.*******:************************: *.****

Human FNDC1        LVDGLQPGERYLFKIRATNRRGLGPHSKAFIVAMPTTSKA--DVEQNTEDNGKPEKPEPS    478
DSS rat Fndc1      LVDGLQPGERYLFKIRATNRRGQGPHSKAFIVAIPTTSSTEASVQPNGRDNGKPEKPQQP    376
                   ********************** **********:****.:  .*: * .********:

Human FNDC1        S-PSPRAPASSQHPSVPASPQGRNAKDLLLDLKNKILANGGAPRKPQLRAKKAEELDLQS    537
DSS rat Fndc1      SSSAPKVAASSQ-----HTPSAKNVKDALSDLKNKIQTNGVAPGRTQLHSK-VGELDPQS    430
                   *  :*:. ****      :*..:*.** * ****** :** ** : **::* . *** **

Human FNDC1        TEITGEEELGSREDSPMSPSDTQDQKRTLRPPSRHGHSVVAPGRTAVRARMPALPRREGV    597
DSS rat Fndc1      TEVTGEEELDSLEDPRSSRLETLNQKQPLRVPSRSGHGALAPGRTPARAGLPVLSRKEGM    490
                   **:******.* **   *  :* :**: ** *** **..:***** .** :*.* *:**:

Human FNDC1        DKPGFSLATQPRPGAPPSASASPAHHASTQGTSHRPSLPASLNDNDLVDSDEDERAVGSL    657
DSS rat Fndc1      DRRGPSLDPHPHPRVEPSASSAYHQLSS--------------TDNDSVDRKEDDQ-AGSP    535
                   *: * **  :*:* . ****::  : :*              .*** ** .**:: .**

Human FNDC1        HPKG--------AFAQPRPALSPSRQSPSSVLRDRSSVHPGAKPASPARR-TPHSGAAEE    708
DSS rat Fndc1      DPKAASSGSSPKNPGRSRPTSAPSRHAASNMLRDKSRVHPGTKAASSSTSRQSHSSTSEE    595
                   .**.          .: **: :***:: *.:***:* ****:* ** :     **.::**

Human FNDC1        DSSASAPPSRLSPPHGGSSRLLPTQPHLSSPLSKGGKDGEDAPATNSNAPSRSTMSSSVS    768
DSS rat Fndc1      DS--SAQPSRHFPLHRGS---------STSPLSRGWKDRQDTHASSSHTTSRTASS----    640
                   **  ** ***  * * **          :****:* ** :*: *:.*:: **:: *

Human FNDC1        SHLSSRTQVSEGAEASDGESHGDGDREDGGRQAEATAQTLRARPASGHFHLLRHKPFAAN    828
DSS rat Fndc1      ---SHPSALTEGSEEEDGGADSDRAAEDTIRRAEATAQIQQTRPGLGHFSLIRNKPFTPH    697
                      *  : ::**:* .** :..*   **   *:******  ::**. *** *:*:***: :

Human FNDC1        GRSPSRFSIGRGPRLQPSSSPQSTVPSRAHPRVPSHSDSHPKLSSGIHGDEEDEKPLPAT    888
DSS rat Fndc1      SRNPNRFPRLRGPRLQPSVSPQSTSASKVLTRSPSLPASHTRPGSDVYGDGEDEEPLPAT    757
                   .*.*.**   ******** *****  *:.  * **   ** : .*.::** ***:*****

Human FNDC1        VVNDHVPSSSRQPISRGWEDLRRSPQRGASLHRKEPIPENPKSTGADTHPQGKYSSLASK    948
DSS rat Fndc1      VINDRTPSYPRHPISGSSDTLRRGPQRGASLYRKEPIPENSKAAGADVPPGGR-SPLSSK    816
                   *:**:.**  *:*** . : ***.*******:******** *::***. * *: * *:**

Human FNDC1        AQDVQQSTDADTEGHSPKAQPGSTDRHASPARPPAARSQQHP--SVPRRMTPGRAPQQQP    1006
DSS rat Fndc1      AQGFQQSTT---DEGAPQTSPASTSRQPSPARPPASRSQPSPGSTVPRRMTPDRSSELS-    872
                   **..****    :  :*::.*.**.*: *******:***  *  :*******.*: : .

Human FNDC1        PPPVATSQHHPGPQSRDAGRSPSQPRLSLTQAGRPRPTSQGRSHSSSDPYTASSRGMLPT    1066
DSS rat Fndc1      -------------SSQSKDRSLSQPKLSVAHAGH------------DHPHTANSRGVLPS    907
                                .*:. .** ***:**:::**:            ..*:**.***:**:
```

```
Human FNDC1       ALQNQDEDAQGSYDDDSTEVEAQDVRAPAHAARAKEAAASLPKHQQVESPTGAGAGGDHR       1126
DSS rat Fndc1     APQNQNEGAQSTYEDNSTEIEGPDSRTPTHSARAKDTTPPILKPRQVGSQSW---SSDNR       964
                  * ***:*.**.:*:*:***:*. * *:*:*:****:::  : * :** * :     ..*:*

Human FNDC1       SQRGHAASPARPSRPGGPQSRARVPSRAAP----GKSEPPSKRPLSSKSQQSVSAEDDEE       1182
DSS rat Fndc1     PQRSQAGASERPIRPGSTHPRAQVPGRAGVQATSVKKVSPSKRPLPLESQQSVFAEEEE-       1023
                   **.:*.:  ** ***. : **:**.**.       *.  ******  :***** **::*

Human FNDC1       EDAGFFKGGKEDLLSSSVPKWPSSSTPRGGKDADGSLAKEEREPAIALAPRGGSLAPVKR       1242
DSS rat Fndc1     ENEGML-KGKEDSLSTSVKKWPSSSSPRDKYA-DRNLDKDK--AAIGLLVQEENT----V       1075
                  *: *::  **** **:** ******:**.     * .* *::   **.*   :   .

Human FNDC1       PLPPPPGSSPRASHVPSRLPPRSAATVSPVAGTHPWPQYTTRAPPGHFSTTPMLSLRQRM       1302
DSS rat Fndc1     PGRRPPGSPAIASHPSTRHQPRNPATASPIANTHSWPRYTTRAPSSYSSTTPMLSLRQRM       1135
                  *   ****   ***   :*  **. **.**:*.** **:******  .: ************

Human FNDC1       MHARFRNPLSRQP--ARPSYRQGYNGRPNVEGKVLPGSNGKPNGQRIINGPQGTKWVVDL       1360
DSS rat Fndc1     -QRRFRTPVSRQPPPPRPVLTPGYNGRPNAEENIPPGSIGKPNGQRIINGPQGTKWVVDL       1194
                   : ***.*:****   **    *******.* :: *** *********************

Human FNDC1       DRGLVLNAEGRYLQDSHGNPLRIKLGGDGRTIVDLEGTPVVSPDGLPLFGQGRHGTPLAN       1420
DSS rat Fndc1     DRGLVLNAEGRYLQDSHGNPLRVRLGGDGRTIVDLGGTPMVSPDGLPLFGQGRHGKPVAS       1254
                  **********************::*********** ***:***************.*:*.

Human FNDC1       AQDKPILSLGGKPLVGLEVIKKTTHPPTTTMQPTTTTTTPLPTTTTPRPTTATTRRTTTTR       1480
DSS rat Fndc1     AQDKPILSLGGKPLVGLEVVRTTTQVPTTTMPPSTTTTTVPPTTTLPPTTTTTTRRTTTTR       1314
                  *******************::.**: ***** *:**** :* ***  ***:*********

Human FNDC1       RTT-TRRPTTTVRTTTR-TTTTTTPTPTTPIPTCPPGTLERHDDDGNLIMSSNGIPECYA       1538
DSS rat Fndc1     RTTTTRRPTTTTRATRRTTTTTTTTPEPTTPSPTCPPGTLEHRDEAGNLIMGSNGIPECYL       1374
                  *** *******.*:* * ******* **** *********::*: *****.********

Human FNDC1       EEDEFSGLETDTAVPTEEAYVIYDEDYEFETSRPPTTTEPSTTATTPRVIPEEGAISSFP       1598
DSS rat Fndc1     EEDDFSGLEIDTALPTEEDYVVYDDDYGLETTRPPTSTMPSTTAATPKVVPEQGTVSSFP       1434
                  ***:***** ***:**** **:**:** :**:****:* *****:**:*:**:*::****

Human FNDC1       EEEFDLAGRKRFVAPYVTYLNKDPSAPCSLTDALDHFQVDSLDEIIPNDLKKSDLPPQHA       1658
DSS rat Fndc1     EEEFDLAGKRRFVAPYVTYLSKDPAAPCSLTDALDHFQVESLDELIPNDLTKNDLPPQHA       1494
                  ********::**********.***:**************:****:*****.*.*******

Human FNDC1       PRNITVVAVEGCHSFVIVDWDKATPGDVVTGYLVYSASYEDFIRNKWSTQASSVTHLPIE       1718
DSS rat Fndc1     PRNITVVAMEGCHSFVIVDWNKAIPGDVVTGYLVYSASYEDFIRNKWSTQTSSVTHLPIE       1554
                  ********:***********:** *************************:*********

Human FNDC1       NLKPNTRYYFKVQAQNPHGYGPISPSVSFVTESDNPLLVVRPPGGEPIWIPFAFKHDPSY       1778
DSS rat Fndc1     NLKPNTRYYFKVQAKNPHGYGPVSPSVSFVTESDNPLLVVRPPGGEPIWIPFAFKHDPGY       1614
                  **************:*******:***********************************.*

Human FNDC1       TDCHGRQYVKRTWYRKFVGVVLCNSLRYKIYLSDNLKDTFYSIGDSWGRGEDHCQFVDSH       1838
DSS rat Fndc1     TDCHGRQYVKRTWYKKFVGVVLCNSLRYKIYLSDNLKDTFYSIGDSWGRGEDHCQFVDSH       1674
                  **************:*********************************************

Human FNDC1       LDGRTGPQSYVEALPTIQGYYRQYRQEPVRFGNIGFGTPYYYVGWYECGVSIPGKW  1894
DSS rat Fndc1     LDGRTGPQSYVEALPTIQGYYRQYRQEPVSFGHIGFGTPYYYVGWYECGVSIPGKW  1730
                  ***************************** **:***********************
```

* indicates amino acid identity (85%). Probable human FNDC1 missense mutations are shaded, which were curated from https://www.ncbi.nlm.nih.gov/snp/?term=fndc1+missense and as of Nov. 12, 2018. Only those missense mutations with minor alleles that were observed at least 2 times (marked in red) in the tested populations are included with the validation status by 1000Genomes. Amino acids in blue indicate that the minor allele has been observed more than 10 times. Rat missense mutations have been experimentally and individually confirmed and are shaded in green. DSS, Dahl salt-sensitive rats.

Table Supplementary S5 (Table S5): **Amino acid alignment and missense mutations in codons for RELT TNF receptor (RELT) in humans and rats**

```
Human_RELT        MKPSLLCRPLSCFLMLLPWPLATLTSTTLWQCPPGEEPDLDPGQGTLCRPCPPGTFSAAW        60
DSS_Rat_Relt      MKRTLLCWPLSCLFVLLPWPLATPTPITPWLCPPGTEPDPDPGQGTLCRTCPPGTFSASL        60
                  ** :*** ****:::******** *  * * **** *** ********* ********: 

Human_RELT        GSSPCQPHARCSLWRRLEAQVGMATRDTLCGDCWPGWFGPWGVPRVPCQPCSWAPLGTHG        120
DSS_Rat_Relt      DSYPCQPHYRCSLQKRLEAQAGTATHDTVCGDCQRGWFGPQGVPHVPCQPCSKATPSTSD        120
                  .* ***** **** :*****.* **:**:****  ***** ***:******* *  .* .

Human_RELT        CDEWGRRARRGVEVAAGASSGGETRQPGNGTRAGGPEETAAQYAVIAIVPVFCLMGLLGI        180
DSS_Rat_Relt      CD--GRRARRGVEVAAGANSNGELRQPGNGTRAGGPEETAAQYAVIAIVPVFCLMGLLGI        178
                  **  **************.*.** ************************************

Human_RELT        LVCNLLKRKGYHCTAHKEVGPGPGGGGSGINPAYRTEDANEDTIGVLVRLITEKKENAAA        240
DSS_Rat_Relt      LVCNLLKRKGYHCTAQKEVGPSPGGGGSGINPAYRTEDANEDTIGVLVRLITEKKENAAA        238
                  ***************:*****.**************************************

Human_RELT        LEELLKEYHSKQLVQTSHRPVSKLPPAPPNVPHICPHRHHLHTVQGLASLSGPCCSRCSQ        300
DSS_Rat_Relt      LEELLKEYHSKQLVQTSHRPVPRLLPASPSTPHVCPHHHHLHTVQGLASLSGPCCSRCSQ        298
                  ********************* :* ** *..**:***:**********************

Human_RELT        KKWPEVLLSPEAVAATTPVPSLLPNPTRVPKAGAKAGRQGEITILSVGRFRVARIPEQRT        360
DSS_Rat_Relt      -KWPEVLLSPEAAAATTPAPTLLPSTSRAPKASAKPGRQGEITILSVGRFRVARIPEQRT        357
                   ***********.*****.*:***. :*.***.** ************************

Human_RELT        SSMVSEVKTITEAGPSWGDLPDSPQPGLPPEQQALLGSGGSRTKWLKPPAENKAEENRYV        420
DSS_Rat_Relt      SSLLSEVKTITEAGPSESDLPESPQPGLPPEQRALLGSSGSHAKWLKPPAENKAEENRYV        417
                  **::************ .***:**********:*****.**::*****************

Human_RELT        VRLSESNLVI 430
DSS_Rat_Relt      VRLSESNLVI 427
                  **********
```

* indicates amino acid identity (88%) between human and the rat. Probable human missense mutations (The Genomes Project et al., 2015) are shaded, which were curated from https://www.ncbi.nlm.nih.gov/snp/ and as of April 23, 2020. Only those missense mutations with minor alleles that were observed at least 2 times (marked in red) in the tested populations are included with the validation status by 1000Genomes. Amino acids in blue indicate that the minor allele has been observed more than 10 times. The rat missense mutation has been experimentally confirmed and shaded in green. DSS, Dahl salt-sensitive rats.

Table Supplementary S6 (Table S6): **Amino acid alignment and missense mutations in codons for uromodulin (UMOD) in humans and rats**

```
Human_UMOD        MG-QPSLTWMLMV-VVASWFITTAATDTSEARWCSECHSNATCTEDEAVTTCTCQEGFTG        58
DSS_Rat_UMOD      MGQLLSLTWLLLVMVVTPWFTVAGANDSPEARRCSECHDNATCVLDGVVTTCSCQAGFTG        60
                  **   ****:*:* **: ** .:.*.*: *** *****.****. * .****:** ****

Human_UMOD        DGLTCVDLDECAIPGAHNCSANSSCVNTPGSFSCVCPEGFRLSPGLGCTDVDECAEPGLS        118
DSS_Rat_UMOD      DGLVCEDIDECATPWTHNCS-NSICMNTLGSYECSCQDGFRLTPGLGCIDVNECTEQGLS        119
                  ***.* *:**** * :**** ** *:** **:.* * :****:***** **:**:* ***

Human_UMOD        HCHALATCVNVVGSYLCVCPAGYRGDGWHCECSPGSCGPGLDCVPEGD--ALVCADPCQA        176
DSS_Rat_UMOD      NCHSLATCVNTEGSYSCVCPKGYRGDGWYCECSPGFCEPGLDCLPQGPSGKLVCQDPCNV        179
                  :**:******. *** **** *******:****** * *****:*:*    *** ***:.

Human_UMOD        HRTLDEYWRSTEYGEGYACDTDLRGWYRFVGQGGARMAETCVPVLRCNTAAPMWLNGTHP        236
DSS_Rat_UMOD      YETLTEYWRSTDYGAGYSCDSDMHGWYRFTGQGGVRMAETCVPVLRCNTAAPMWLNGSHP        239
                  :.** ******:** **:**:*::*****.****.**********************:**

Human_UMOD        SSDEGIVSRKACAHWSGHCCLWDASVQVKACAGGYYVYNLTAPPECHLAYCTDPSSVEGT        296
DSS_Rat_UMOD      SSREGIVSRTACAHWSDHCCLWSTEIQVKACPGGFYVYNLTEPPECNLAYCTDPSSVEGT        299
                  ** ******.******.*****.:.:***** **:****** ****:*************

Human_UMOD        CEECSIDEDCKSNNGRWHCQCKQDFNITDISLLEHRLECGANDMKVSLGKCQLKSLGFDK        356
DSS_Rat_UMOD      CEECGVDEDCVSDNGRWRCQCKQDFNVTDVSLLEHRLECEANEIKISLSKCQLQSLGFMK        359
                  ****.:**** *:****:*******:**:********* **::*:**.****:**** *
```

```
Human_UMOD        VFMYLSDSRCSGFNDRDNRDWVSVVTPARDGPCGTVLTRNETHATYSNTLYLADEIIIRD    416
DSS_Rat_UMOD      VFMYLNDRQCSGFSERGERDWMSIVTPARDGPCGTVLRRNETHATYSNTLYLASEIIIRD    419
                  *****.* :****.:*.:***:*:************* ***************.******

Human_UMOD        LNIKINFACSYPLDMKVSLKTALQPMVSALNIRVGGTGMFTVRMALFQTPSYTQPYQGSS    476
DSS_Rat_UMOD      INIRINFECSYPLDMKVSLKTSLQPMVSALNISLGGTGKFTVQMALFQNPTYTQPYQGPS    479
                  :**:*** *************:********** :**** ***:*****.*:******* *

Human_UMOD        VTLSTEAFLYVGTMLDGGDLSRFALLMTNCYATPSSNATDPLKYFIIQDRCPHTRDSTIQ    536
DSS_Rat_UMOD      VMLSTEAFLYVGTMLDGGDLSRFVLLMTNCYATPSSNSTDPVKYFIIQDRCPHTEDTTIQ    539
                  * *********************.*************:***:************.*:***

Human_UMOD        VVENGESSQGRFSVQMFRFAGNYDLVYLHCEVYLCDTMNEKCKPTCSGTRFRSGSVIDQS    596
DSS_Rat_UMOD      VTENGESSQARFSIQMFRFAGNSDLVYLHCEVYLCDTMSEQCKPTCSGTRYRSGNFIDQT    599
                  *.*******.***:******** ***************.*:*********:***..***:

Human_UMOD        RVLNLGPITRKGVQATVSRAF-SSLGLLKVWLPLLLSATLTLTFQ    640
DSS_Rat_UMOD      RVLNLGPITRQGVQASVSKAASSNLGFLSIWLLLFLSATLTLMVH    644
                  **********:****:**:*  *.**:*.:** *:******* .:
```

* indicates amino acid identity (81%) between human and the rat. Probable human missense mutations (The Genomes Project et al., 2015) are shaded, which were curated from https://www.ncbi.nlm.nih.gov/snp/ and as of April 23, 2020. Only those missense mutations with minor alleles that were observed at least 2 times (marked in red) in the tested populations are included with the validation status by 1000Genomes. Amino acids in blue indicate that the minor allele has been observed more than 10 times. The rat missense mutation has been experimentally confirmed and shaded in green. DSS, Dahl salt-sensitive rats.

Table Supplementary S7 (Table S7): Amino acid alignment and missense mutations in codons for BAG cochaperone 3 (BAG3) in humans and rats

```
Human_BAG3        MSAATHSPMMQVASGNG--DRDPLPPGWEIKIDPQTGWPFFVDHNSRTTTWNDPRVPSEG    58
DSS_Rat_Bag3      MSAATQSPVMQMASGNGASDRDPLPPGWEIKIDPQTGWPFFVDHNSRTTTWNDPRVPPEG    60
                  *****:**:**:*****  ************************************** **

Human_BAG3        PKETPSSANGPSREGSRLPPAREGHPVYPQLRPGYIPIPVLHEGAENRQVHPFHVYPQPG    118
DSS_Rat_Bag3      PKETASSANGPSRDGSRLLPAREGHPIYPQLRPGYIPIPVHHEGSENRQPHLFHAYSQPG    120
                  **** ********:**** *******:************* ***:**** * **.* ***

Human_BAG3        MQRFRTEAAAAAPQRSQSPLRG-MPETTQPDKQCGQVAAAAAAQPPASHGPERSQSPAAS    177
DSS_Rat_Bag3      VQRFRTEAAAAAPQRSQSPLRGGVTETTQTDKQCGQVPAAATAQPPTAHGPERSQSPAAS    180
                  :********************* : **** ******* ***:****::************

Human_BAG3        DCSSSSSSASLPSSGRSSLGSHQLPRGYISIPVIHEQNVTRPAAQPSFHQAQKTHYPAQQ    237
DSS_Rat_Bag3      DCSSSSSSASLPSSGRSSLGSHQLPRGYIPIPVIHEQNITRPAAQPSFHQAQKTHYPAQQ    240
                  ***************************** ********:*********************

Human_BAG3        GEYQTHQPVYHKIQGDDWEPRPLRAASPFRSSVQGASSREGSPARSSTPLHSPSPIRVHT    297
DSS_Rat_Bag3      GEYQPQQPVYHKIQGDDWEPRPLRATSPFRSPVRGASSREGSPARSGTPVHCPSPIRVHT    300
                  **** :*******************:***** *:************.**:*.********

Human_BAG3        VVDRPQQPMTHRETAPVSQPENKPESKPGPVGPELPPGHIPIQVIRKEVDSKPVSQKPPP    357
DSS_Rat_Bag3      VVDRP-QPMTHREPPPVTQPENKPESKPGLAGSDLPPGHIPIQVIRREADSKPVSQKPPP    359
                  ***** *******  **:*********** .* :************:*.***********

Human_BAG3        PSEKVEVKVPPAPVPCPPPSPGPSAVPSSPKSVATEERAAPSTAPAEATPPKPGEAEAPP    417
DSS_Rat_Bag3      PAEKVEVKVSSAPIPCPSPGPAPSAVPSSPKNVAAEPKAAPSPAPAEAAPLKSGDAEAPP    419
                  *:*******  **:*** *.*.*********.**:* :**** *****:* * *:*****

Human_BAG3        KHPGVLKVEAILEKVQGLEQAVDNFEGKKTDKKYLMIEEYLTKELLALDSVDPEGRADVR    477
DSS_Rat_Bag3      KHPGVLKVEAILEKVQGLEQAVDNFEGKKTDKKYLMIEEYLTKELLALDSVDPEGRADVR    479
                  ************************************************************

Human_BAG3        QARRDGVRKVQTILEKLEQKAIDVPGQVQVYELQPSNLEADQPLQAIMEMGAVAADKGKK    537
DSS_Rat_Bag3      QARRDGVRKVQTILEKLEQKAIDVPGQVQVYELQPSNLEPEQPLQEIM--GAVAADKDKK    537
                  *************************************** :**** **  *******.**

Human_BAG3        NAGNAEDPHTETQQPEATAAATSNPSSMTDTPGNPAAP 575
DSS_Rat_Bag3      G-PENEDPQTESQQLEAKAATPPNPSSTADSAGNPVAP 574
                  .  : ***:**:** **.**:  **** :*: ***.**
```

* indicates amino acid identity (89%) between human and the rat. Probable human missense mutations (The Genomes Project et al., 2015) are shaded, which were curated from https://www.ncbi.nlm.nih.gov/snp/ and as of April 23, 2020. Amino acids in blue indicate that the minor allele has been observed more than 10 times. The rat missense mutation has been experimentally confirmed and shaded in green. Human 455K was associated in a GWAS with heart failure due to dilated cardiomyopathy (Eur Heart J. 2011;32:1065). DSS, Dahl salt-sensitive rats.

Table Supplementary S8 (Table S8): **Amino acid alignment and missense mutations in codons for COBW domain containing (CBWD1) in humans and rats**

```
Human_CBWD1        MLPAVGSADEEEDPAEEDCPELVPIETTQSEEEEKSGLGAKIPVTIITGYLGAGKTTLLN      60
DSS_Rat_Cbwd1      MLPAMKTVEA-EEEYAEDCPELVPIETKHQEKEENLDFIIKIPVTIVTGYLGAGKTTLLN      59
                   ****: :.:  *:   ***********.:.*:**: .:  ******:*************

Human_CBWD1        YILTEQHSKRVAVILNEFGEGSALEKSLAVSQGGELYEEWLELRNGCLCCSVKDSGLRAI      120
DSS_Rat_Cbwd1      YILTEQHNRKIAVILNEFGEGNAVEKSLAVSQGGELYEEWLELRNGCLCCSVKDNGLKAI      119
                   *******.:::**********.*:******************************.**:**

Human_CBWD1        ENLMQKKGKFDYILLETTGLADPGAVASMFWVDAELGSDIYLDGIITIVDSKYGLKHLTE      180
DSS_Rat_Cbwd1      ENLMQKKGKFDYILLETTGLADPGAVASMFWVDAELGSDIYLDGIITVVDSKYGLKHLTE      179
                   ***********************************************:************

Human_CBWD1        EKPDGLINEATRQVALADAILINKTDLVPEEDVKKLRTTIRSINGLGQILETQRSRVDLS      240
DSS_Rat_Cbwd1      EKPDGLVNEATRQVALADMILINKTDLVSEEELNKLRTTIRSINGLGKVLETQRSRTHLS      239
                   ******:*********** ********* **:::*************::*******..**

Human_CBWD1        NVLDLHAFDSLSGISLQKKLQHVPGTQPHLDQSIVTITFEVPGNAKEEHLNMFIQNLLWE      300
DSS_Rat_Cbwd1      NILDLHAYDTLSGISLQKKLQHVS-TAPHLDQSIVTVTFDVPGSAEEESLNVFIQNLLWE      298
                   *:*****:*:************   * *********:**:***.*:** **:********

Human_CBWD1        KNVRNKDNHCMEVIRLKGLVSIKDKSQQVIVQGVHELYDLEETPVSWKDDTERTNRLVLL      360
DSS_Rat_Cbwd1      KNVKNKDGRCMEVIRLKGLVSIKDKPQQMIVQGIHELYELEESRVNWKDDAERACQLVFI      358
                   ***:***.:**************** **:****:****:***: *.****:**: :**::

Human_CBWD1        GRNLDKDILKQLFIATVTETEKQWTTRFQEDQVCT--  395
DSS_Rat_Cbwd1      GKNLDKDILQQLFITAVAETKEQTTAP-GQDGACPPH  394
                   *:*******:****::*:**::* *:    :* .*
```

* indicates amino acid identity (85%) between human and the rat. Probable human missense mutations (The Genomes Project et al., 2015) are shaded, which were curated from https://www.ncbi.nlm.nih.gov/snp/ and as of April 23, 2020. Only those missense mutations with minor alleles that were observed at least 2 times (marked in red) in the tested populations are included with the validation status by 1000Genomes. Amino acids in blue indicate that the minor allele has been observed more than 10 times. The rat missense mutation has been experimentally confirmed and shaded in green. DSS, Dahl salt-sensitive rats.

Table Supplementary S9 (Table S9): **Amino acid alignment and missense mutations in codons for dedicator of cytokinesis 8 (DOCK8) in humans and rats**

```
Human_DOCK8        MATLPSAERRAFALKINRYSSAEIRKQFTLPPNLGQYHRQSISTSGFPSLQLPQFYDPVE      60
DSS_Rat_Dock8      MATLPSAERRAFALKINRYSSSEIRKQFTLPPNLGQYHRHSLSTSGFPSLQLPQLYEPVE      60
                   *********************:*****************:*:************:*:***

Human_DOCK8        PVDFEGLLMTHLNSLDVQLAQELGDFTDDDLDVVFTPKECRTLQPSLPEEGVELDPHVRD      120
DSS_Rat_Dock8      PVDFEGLVMTHLNSLDAELAQELGDLTDDDLHVAFTPKECRTLQHSLPEEGVELDPHVRD      120
                   *******:********.:*******:*****.*.********** ***************

Human_DOCK8        CVQTYIREWLIVNRKNQGSPEICGFKKTGSRKDFHKTLPKQTFESETLECSEPAAQAGPR      180
DSS_Rat_Dock8      CVQTYIREWLIVNRKNQGSPEFCSFKKTGSRRDFHKTLQKQTFESETLECSEPATQTGPR      180
                   *********************:*.*******:****** ***************:*:***

Human_DOCK8        H-LNVLCDVSGKGPVTACDFDLRSLQPDKRLENLLQQVSAEDFEKQNEEARRTNRQAELF      239
DSS_Rat_Dock8      HRFNVLCDVSGKGPITSCDFDLRSLQPDERLENLLQ-LSAEDFEKEKEEARKTNRTAELF      239
```

```
                         * :***********:*:***********:******* :*******::****:*** ****

Human_DOCK8              ALYPSVDEEDAVEIRPVPECPKEHLGNRILVKLLTLKFEIEIEPLFASIALYDVKERKKI        299
DSS_Rat_Dock8            ALYPSVDEEDAVEIRPVPECPKEHLGNRILVKVLTLKFEIEIEPLFASIALYDVKERKKI        299
                         ********************************:***************************

Human_DOCK8              SENFHCDLNSDQFKGFLRAHTPSVAASSQARSAVFSVTYPSSDIYLVVKIEKVLQQGEIG        359
DSS_Rat_Dock8            SENFHCDLNSDQFKGFLRAHTPSVDPSSQARSAVFSVTYPSSDIYLVVKIEKVLQQGEIA        359
                         ************************  *********************************.

Human_DOCK8              DCAEPYTVIKESDGGKSKEKIEKLKLQAESFCQRLGKYRMPFAWAPISLSSFFNVSTLER        419
DSS_Rat_Dock8            DCAEPYMIIKESDGGKSKEKVEKLKLQAESFCQRLGKYRMPFAWAPISLASFFNVSTLER        419
                         ****** :************:****************************:**********

Human_DOCK8              EVTDVDSVVGRSSVGERRTLAQSRRLSERALSLEENGVGSNFKTSTLSVSSFFKQEGDRL        479
DSS_Rat_Dock8            ESTDLEPGAGRNSVGERRSLSQSRRPSERTLSFEENGVGSNFKTTTLATNIFFKQEGDRL        479
                         * **::  .**.******:*:**** ***:**:***********:**:.. *********

Human_DOCK8              SDEDLFKFLADYKRSSSLQRRVKSIPGLLRLEISTAPEIINCCLTPEMLPVKPFPENRTR        539
DSS_Rat_Dock8            SDEDLFKFLADYKRSSSLQRRVKSIPGSLRLEIAPAPDVMNCCLTPEMLPVKPFPENRTR        539
                         *************************** *****: **:::********************

Human_DOCK8              PHKEILEFPTREVYVPHTVYRNLLYVYPQRLNFVNKLASARNITIKIQFMCGEDASNAMP        599
DSS_Rat_Dock8            PHKEILEFPIREVYVPHTVYRNLLYVYPQRLNFANKLASARNITIKIQFMCGEDPSNAMP        599
                         ********* ***********************.******************** *****

Human_DOCK8              VIFGKSSGPEFLQEVYTAVTYHNKSPDFYEEVKIKLPAKLTVNHHLLFTFYHISCQQKQG        659
DSS_Rat_Dock8            VIFGKSSGPEFLQEVYTAITYHNKSPDFYEEVKIKLPAKLTVNHHLLFTFYHISCQQKQG        659
                         ******************:*****************************************

Human_DOCK8              ASVETLLGYSWLPILLNERLQTGSYCLPVALEKLPPNYSMHSAEKVPLQNPPIKWAEGHK        719
DSS_Rat_Dock8            ASGESLLGYSWLPILLNERLQTGSYCLPVALEKLPPNYSIHSAEKVPLQNPPIKWAEGHK        719
                         ** *:**********************************:********************

Human_DOCK8              GVFNIEVQAVSSVHTQDNHLEKFFTLCHSLESQVTFPIRVLDQKISEMALEHELKLSIIC        779
DSS_Rat_Dock8            GVFNIEVQAVSSVHTQDNHLEKFFTLCHSLESQVTFPIRVLDQKITENTLEHELKLSIIC        779
                         *********************************************:* :***********

Human_DOCK8              LNSSRLEPLVLFLHLVLDKLFQLSVQPMVIAGQTANFSQFAFESVVAIANSLHNSKDLSK        839
DSS_Rat_Dock8            LNSSRLEPLVLFLHLVLDKLFQLSVQPMVIAGQTANFSQFAFESVVAIANSLHNSKDLRK        839
                         ********************************************************** *

Human_DOCK8              DQHGRNCLLASYVHYVFRLPEVQRDVPKSGAPTALLDPRSYHTYGRTSAAAVSSKLLQAR        899
DSS_Rat_Dock8            DQHGRNCLLASYVHYVFRLPELHRDAPKSGGPTAVVPDPRYHTYGRTSAAAVSSKLMQAR        899
                         *********************::**.****.***::    ****************:***

Human_DOCK8              VMSSSNPDLAGTHSAADEEVKNIMSSKIADRNCSRMSYYCSGSSDAPSSPAAPRPASKKH        959
DSS_Rat_Dock8            VMSSSNPDLAGTHCAADEEVKNIMSSKIADRNCSRMSYYCSGNSDVPGSTAAPRPVSKKH        959
                         *************.****************************.**.*.* *****.****

Human_DOCK8              FHEELALQMVVSTGMVRETVFKYAWFFFELLVKSMAQHVHNMDKRDSFRRTRFSDRFMDD        1019
DSS_Rat_Dock8            FHEELALQMVVSTGLVRETVFKYAWFFFELLVKSMAQYVHNLDKRDSFRRTRFSDRFKDD        1019
                         **************:**********************:***:*************** **

Human_DOCK8              ITTIVNVVTSEIAALLVKPQKENEQAEKMNISLAFFLYDLLSLMDRGFVFNLIRHYCSQL        1079
DSS_Rat_Dock8            ITTIVNVVTSEIAALLVKPQKESEQAEKINISLAFFLYDLLSIMDRGFVFNLIKHYCTQL        1079
                         **********************.*****:*************:**********:***:**

Human_DOCK8              SAKLSNLPTLISMRLEFLRILCSHEHYLNLNLFFMNADTAPTSPCPSISSQNSSSCSSFQ        1139
DSS_Rat_Dock8            SAKLNTLPTLISMRLEFLRILCSHEHYLNLNLLFMNPDTAPASPCPSISSQNSSSCSSFQ        1139
                         ****..**************************:*** ****:******************

Human_DOCK8              DQKIASMFDLTSEYRQQHFLTGLLFTELAAALDAEGEGISKVQRKAVSAIHSLLSSHDLD        1199
DSS_Rat_Dock8            DQKIASMFDLTPEYRQQHFLTGLLFTELAVALDAEGDGISRVQRKAVSAIHSLLSSHDLD        1199
                         *********** *****************.******:***:*******************

Human_DOCK8              PRCVKPEVKVKIAALYLPLVGIILDALPQLCDFTVADTRRYRTSGSDEEQEGAGAINQNV        1259
DSS_Rat_Dock8            PRCLKPEVKVKIAALYLPLVGIILDALPQLYDFTDARSGRSRASGSYEEQDVANGINQNV        1259
                         ***:************************** *** * : * *:*** ***: *..*****

Human_DOCK8              ALAIAGNNFNLKTSGIVLSSLPYKQYNMLNADTTRNLMICFLWIMKNADQSLIRKWIADL        1319
DSS_Rat_Dock8            ALAIAGNHFNLKTSGAMLSSLPYKQYNMLNADTTRHLMVCFLWIMKNADQSLIRKWIADL        1319
                         *******:******* :******************:**:*********************

Human_DOCK8              PSTQLNRILDLLFICVLCFEYKGKQSSDKVSTQVLQKSRDVKARLEEALLRGEGARGEMM        1379
DSS_Rat_Dock8            PSMQLNRILDLLFICVSCFEYKGKQSSDKVSNQVLQKSRDVKAKLEEALLRGEGARGEMM        1379
```

```
                         ** ************* ************** .*********** :****************

Human_DOCK8              RRRAPGNDRFPGLNENLRWKKEQTHWRQANEKLDKTKAELDQEALISGNLATEAHLIILD        1439
DSS_Rat_Dock8            RRRIPGTDRFPGLNENLRWRKEQTQWRQANEKLDKTKAELDQEALISGNLATEANLIILD        1439
                         *** **.************:****:*****************************:*****

Human_DOCK8              MQENIIQASSALDCKDSLLGGVLRVLVNSLNCDQSTTYLTHCFATLRALIAKFGDLLFEE        1499
DSS_Rat_Dock8            MQENIIQASSSLDCKDSLLGGVLRVLVNSLSCDQSTTYLTHCFATLRALIAKFGDLLFEE        1499
                         **********:*******************.*****************************

Human_DOCK8              EVEQCFDLCHQVLHHCSSSMDVTRSQACATLYLLMRFSFGATSNFARVKMQVTMSLASLV        1559
DSS_Rat_Dock8            EMEQCADLCQRVLHHCSSSMDVTRSQACATLYLLMRFSFGATSNFARVKMQVTMALASLV        1559
                         *:*** ***::*******************************************:*****

Human_DOCK8              GRAPDFNEEHLRRSLRTILAYSEEDTAMQMTPFPTQVEELLCNLNSILYDTVKMREFQED        1619
DSS_Rat_Dock8            GKAPDFNEEHLRKSLRTILAYSEEDTAMRATLFPTQVEELLCNLNSILYDTVKMREFQED        1619
                         *:**********:***************: * ****************************

Human_DOCK8              PEMLMDLMYRIAKSYQASPDLRLTWLQNMAEKHTKKKCYTEAAMCLVHAAALVAEYLSML        1679
DSS_Rat_Dock8            PEMLMDLMYRIAKSYQASPDLRLTWLQNMAEKHTKRKCFTEAAMCLVHAAALVAEYLSML        1679
                         ***********************************:**:*********************

Human_DOCK8              EDHSYLPVGSVSFQNISSNVLEESVVSEDTLSPDEDGVCAGQYFTESGLVGLLEQAAELF        1739
DSS_Rat_Dock8            EDHSYLPVGSVSFQNISSNVLEESAVSDDTLSPDEDGVCSGRYFTESGLVGLLEQAAELF        1739
                         ************************.**:***********:*:******************

Human_DOCK8              STGGLYETVNEVYKLVIPILEAHREFRKLTLTHSKLQRAFDSIVNKDHKRMFGTYFRVGF        1799
DSS_Rat_Dock8            STGGLYETVNEVYKLVIPILEAHRDFRKLTSTHDKLQKAFDNIINKDHKRMFGTYFRVGF        1799
                         ************************:***** **.***:***.*:****************

Human_DOCK8              FGSKFGDLDEQEFVYKEPAITKLPEISHRLEAFYGQCFGAEFVEVIKDSTPVDKTKLDPN        1859
DSS_Rat_Dock8            YGSKFGDLDEQEFVYKEPAITKLPEISHRLEGFYGQCFGAEFVEVIKDSTPVDKTKLDPN        1859
                         :******************************.****************************

Human_DOCK8              KAYIQITFVEPYFDEYEMKDRVTYFEKNFNLRRFMYTTPFTLEGRPRGELHEQYRRNTVL        1919
DSS_Rat_Dock8            KAYIQITFVEPYFDEYEMKDRVTYFEKNFNLRRFMYTTPFTLEGRPRGELHEQHRRNTVL        1919
                         *****************************************************:******

Human_DOCK8              TTMHAFPYIKTRISVIQKEEFVLTPIEVAIEDMKKKTLQLAVAINQEPPDAKMLQMVLQG        1979
DSS_Rat_Dock8            TTMHAFPYIKTRIRVSQKEEFVLTPIEVAIEDMKKKTLQLAVATHQEPPDAKMLQMVLQG        1979
                         ************* * *************************** :***************

Human_DOCK8              SVGATVNQGPLEVAQVFLAEIPADPKLYRHHNKLRLCFKEFIMRCGEAVEKNKRLITADQ        2039
DSS_Rat_Dock8            SVGATVNQGPLEVAQVFLAEIPADPKLYRHHNKLRLCFKEFIMRCGEAVEKNRRLITAEQ        2039
                         ****************************************************:*****:*

Human_DOCK8              REYQQELKKNYNKLKENLRPMIERKIPELYKPIFRVESQKRDSFHRSSFRKCETQLSQGS        2099
DSS_Rat_Dock8            REYQQELKKNYNRLKDSLRPMIERKIPELYKPIFRVDSQKRDSFHRSSFRKCETQLSQGS        2099
                         ************:**:.*******************:***********************
```

* indicates amino acid identity (94%) between human and the rat. Probable human missense mutations (The Genomes Project et al., 2015) are shaded, which were curated from https://www.ncbi.nlm.nih.gov/snp/ and as of April 23, 2020. Only those missense mutations with minor alleles that were observed at least 2 times (marked in red) in the tested populations are included with the validation status by 1000Genomes. Amino acids in blue indicate that the minor allele has been observed more than 10 times. The rat missense mutation has been experimentally confirmed and shaded in green. DSS, Dahl salt-sensitive rats.

Table Supplementary S10 (Table S10): Amino acid alignment and missense mutations in codons for KN motif and ankyrin repeat domains 1 (KANK1) in humans and rats

```
Human_KANK1              MAHTTKVNGSASGKAGDILSGDQDKEQKDPYFVETPYGYQLDLDFLKYVDDIQKGNTIKR        60
DSS_Rat_Kank1            MAYTTKVNGCAPENADGVLNGDRNKEKKDPYFVETPYGFQLDLDFVKYVDDIQKGNTIKK        60
                         **:******.*  :*..:*.**::**:***********:******:*************:

Human_KANK1              LNIQKRRKPSVPCPEPRTTSGQQGIWTSTESLSSSNSDDNKQCPNFLIARSQVTSTPISK        120
DSS_Rat_Kank1            LNIQKRRKPSGPCAEVRAIPGPQGVWTSTESLSSSNSDDSKQCPSFLLARSHVTSTPIPR        120
                         ********** ** * *:  * **:**************.****.**:***:****** :

Human_KANK1              PPPPLETSLPFLTIPENRQLPPPSPQLPKHNLHVTKTLMETRRRLEQERATMQMTPGEFR        180
```

```
DSS_Rat_Kank1      PPAPLETSPTFAISENRQLLPPPSPQLPRHNLHVTKTLMETRRRLEQERVTMQMAPGEFR      180
                   ** *****  *    :.: *********:********************.****:*****

Human_KANK1        RPRLASFGGMGTTSSLPSFVGSGNHNPAKHQLQNGYQGNGDYGSYAPAAPTTSSMGSSIR      240
DSS_Rat_Kank1      RPRLASFGGMGSTSSLPSFMGSGNHSSAMHQLQNGYQGNGDYSSYVPAVPTTSSMGSSVR      240
                   ***********:*******:*****. * *************.**.**.*********:*

Human_KANK1        HSPLSSGISTPVTNVSPMHLQHIREQMAIALKRLKELEEQVRTIPVLQVKISVLQEEKRQ      300
DSS_Rat_Kank1      HSPLSSGISTPVTNVSPMHLQHIREQMAIALKRLKELEEQVRTIPVLQVKISVLQEEKRQ      300
                   ************************************************************

Human_KANK1        LVSQLKNQRAASQINVCGVRKRSYSAGNASQLEQLSRARRSGGELYIDYEEEEMETVEQS      360
DSS_Rat_Kank1      LASQLKNQRAASQNEACGVRKRSYSAGNASQLELLSRARRGGGELYIDYEEEEMESVEQS      360
                   *.*********** :.***************** ******.**************:****

Human_KANK1        TQRIKEFRQLTADMQALEQKIQDSSCEASSELRENGECR-----SVAVGAEENMNDIVVY      415
DSS_Rat_Kank1      TQRIREFRQLTADMQALEQKIQDSSCEATPELRENGQCASRERKSVAVGSDENMNDIVVY      420
                   ****:***********************: ******:*      *****::*********

Human_KANK1        HRGSRSCKDAAVGTLVEMRNCGVSVTEAMLGVMTEADKEIELQQQTIESLKEKIYRLEVQ      475
DSS_Rat_Kank1      HRDFRSRKDTAVGTVTETRNFGISVTEAMLGVITEADKEIELQQQTIEALKEKIYRLEVQ      480
                   **. ** **:****:.* ** *:*********:***************:***********

Human_KANK1        LRETTHDREMTKLKQELQAAGSRKKVDKATMAQPLVFSKVVEAVVQTRDQMVGSHMDLVD      535
DSS_Rat_Kank1      LKETTHDREMTKLKQELQAAGSRKKVDKAIMAQPLVFSKLVEALVPTREQMVGSHVDTTD      540
                   *:*************************** *********:***:* **:******:* .*

Human_KANK1        TCVGTSVETNSVGISCQPECKNKVVGPELPMNWWIVKERVEMHDRCAGRSVEMCDKSVSV      595
DSS_Rat_Kank1      SCVGTSVQTSSVGTSCHPDRKNQVVGPELPMNWWVVKERVGTHDRCVGRSVETCNRSVGV      600
                   :******:*.*** **:*: **:***********:*****  ****.***** *::**.*

Human_KANK1        EVSVCETGSNTEESVNDLTLLKTNLNLKEVRSIGCGDCSVDVTVCSPKECASRGVNTEAV      655
DSS_Rat_Kank1      EVSVCETGSNTEASGSDLTLLKTNLNLKDVRSIGCGDCSVDVIVCLPKECTSRSMNTEAV      660
                   ************ * .************:************* ** ****:**.:*****

Human_KANK1        SQVEAAVMAVPRTADQDTSTDLEQVHQFTNTETATLIESCTNTCLSTLDKQTSTQTVETR      715
DSS_Rat_Kank1      GQGEAAVMAVPHTTDQHTSTNLERVDQGTNTEVTTLVESCTNTLLSTLDKQTSTQNVEMR      720
                   .* ********:*:**.***:**:*.* ****.:**:****** ***********.** *

Human_KANK1        TVAVGEGRVKDINSSTKTRSIGVGTLLSGHSGFDRPSAVKTKESGVGQININDNYLVGLK      775
DSS_Rat_Kank1      TVAIGEGRVRDINPSTKTRSVGVGTVLSGSSDFDKPCAVKTKESGVGQINIHDNYLVGLK      780
                   ***:*****:*** ******:****:*** *.**:*.**************:********

Human_KANK1        MRTIACGPPQLTVGLTASRRSVGVGDDPVGESLENP-QPQAPLGMMTGLDHYIERIQKLL      834
DSS_Rat_Kank1      MRTIACGPPQLTVGLIGSRRSVGVGNEPVGDLLEDPLQPQVRSGMMTGLDHYIERVQKLL      840
                   *************** .********::***: **:* ***.  ************:****

Human_KANK1        AEQQTLLAENYSELAEAFGEPHSQMGSLNSQLISTLSSINSVMKSASTEELRNPDFQKTS      894
DSS_Rat_Kank1      AEQQTLLAENYSELAEAFGEPHSQIGSLNSQLISTLSSINSVMKSASTEELRNPDFQKSS      900
                   ************************:*********************************:*

Human_KANK1        LGKITGNYLGYTCKCGGLQSGSPLSSQTSQPEQEVGTSEGKPISSLDAFPTQEGTLSPVN      954
DSS_Rat_Kank1      LGKIAGNHLECTCKCGGLRSGGLVNVQTSQPEVETETTEGKR--GCEQFLTQGNTLPPVN      958
                   ****:**:*  *******:**. :. ****** *. *:***   . : * ** .** ***

Human_KANK1        LTDDQIAAGLYACTNNESTLKSIMKKKDGNKDSNGAKKNLQFVGINGGYETTSSDDSSSD      1014
DSS_Rat_Kank1      LTDDQIATGLYVCPNNENTLKSIMKKSDGNKDSNGAKKNLQFIGVNGGYETTSSDDSSSG      1018
                   *******:***.* ***.********.***************:*:**************.

Human_KANK1        ESSSSESDDECDVIEYPLEEEE----EEEDEDTRGMAEGHHAVNIEGLKSARVEDEMQVQ      1070
DSS_Rat_Kank1      GSSSSESDDECDTNGYPPEEEEEEEEEEEEDHDTRGMAEGRHAVNIEGFQSARVEDEVQVP      1078
                    ***********.  ** ****    ****.********:*******::*******:** 

Human_KANK1        ECEPEKVEIRERYELSEKMLSACNLLKNTINDPKALTSKDMRFCLNTLQHEWFRVSSQKS      1130
DSS_Rat_Kank1      ECEPEKEEIRERYELSEKMLSACSLLKYNINDPKALASKDMRICLNTLQHEWFRVSSQKS      1138
                   ****** ****************.*** .*******:*****:*****************

Human_KANK1        AIPAMVGDYIAAFEAISPDVLRYVINLADGNGNTALHYSVSHSNFEIVKLLLDADVCNVD      1190
DSS_Rat_Kank1      AVPAMVGDYIAAFEAVSPDVLRYIINMADGNGNTALHYSVSHSNFQIVKLLLDADVCNVD      1198
                   *:*************:*******:**:******************:**************

Human_KANK1        HQNKAGYTPIMLAALAAVEAEKDMRIVEELFGCGDVNAKASQAGQTALMLAVSHGRIDMV      1250
DSS_Rat_Kank1      HQNKAGYTPIMLAALAAVEAEKDMQVVEELFSCGDVNAKASQAGQTALMLAVSHGRIDMV      1258
                   ************************::*****.****************************

Human_KANK1        KGLLACGADVNIQDDEGSTALMCASEHGHVEIVKLLLAQPGCNGHLEDNDGSTALSIALE      1310
```

```
DSS_Rat_Kank1       KGLLACGADVNIQDDEGSTALMCASEHGHVEIVKLLLAQPGCNGHLEDNDGSTALSIALE        1318
                    ************************************************************

Human_KANK1         AGHKDIAVLLYAHVNFAKAQSPGTPRLGRKTSPGPTHRGSFD      1352
DSS_Rat_Kank1       AGHKDIAVLLYAHLNFSKAQSPSTPRLGRKTSPGPTHRGSFD      1360
                    *************:**:*****.*******************
```

* indicates amino acid identity (87%) between human and the rat. Probable human missense mutations (The Genomes Project et al., 2015) are shaded, which were curated from https://www.ncbi.nlm.nih.gov/snp/ and as of April 23, 2020. Only those missense mutations with minor alleles that were observed at least 2 times (marked in red) in the tested populations are included with the validation status by 1000Genomes. Amino acids in blue indicate that the minor allele has been observed more than 10 times. The rat missense mutation has been experimentally confirmed and shaded in green. DSS, Dahl salt-sensitive rats.

Evangelou, E., Warren, H.R., Mosen-Ansorena, D., Mifsud, B., Pazoki, R., Gao, H., Ntritsos, G., Dimou, N., Cabrera, C.P., Karaman, I., *et al.* (2018). Genetic analysis of over 1 million people identifies 535 new loci associated with blood pressure traits. Nature Genetics *50*, 1412-1425.

The Genomes Project, C., Auton, A., Abecasis, G.R., Altshuler, D.M., Durbin, R.M., Abecasis, G.R., Bentley, D.R., Chakravarti, A., Clark, A.G., Donnelly, P., *et al.* (2015). A global reference for human genetic variation. Nature *526*, 68.
