# Supplementary material for: Shifting Paradigm from Gene Expressions to Pathways Reveals Physiological Mechanisms in Blood Pressure Control in Causation
Source: Int J Mol Sci. 2023 Jan 9;24(2):1262. doi: 10.3390/ijms24021262 (PMC9863686; doi:10.3390/ijms24021262)
Supplement: Supplementary file 1 [file ijms-2062741-author response to first round review.pdf › ijms-2062741-author response to first round review.pdf]

A published resubmitted manuscript (ijms-2062741) from a previously reviewed and rejected manuscript ((ijms-1978980)

Dear Editor of IJMS,

I'm submitting this letter to you as a supplement to our published work (ijms-2062741). The following are our responses to published reviewer comments in the first round of peer review for a fairness of the reviewer process.

Thanks,
Alan Deng

Comments of Reviewer #1:

The topic of the manuscript concerns genetic pathways in the pathophysiology of hypertension and follows the previous works of the authors. All parts of the manuscript are adequate to the solved problem, it is appropriate to organize the manuscript in the form of Introduction, Objective, Methodology, Statistical analysis, Results, Discussion, Conclusion.
Response to Reviewer #1 general comments: Thank you for your appreciations. According to your suggestions on reorganize, we have added new sections as follows: Objective after introduction, and statistical analysis in Method section.

I have several comments:

In the Introduction part: I recommend including the definition of hypertension and severity of hypertension.
Response to Reviewer #1 comments on introduction: Following your recommendation, we have added the definition and severity of hypertension in the first paragraph of Introduction.

Reviewer #1 comments on Methods: Despite the fact that the authors provide citations of their works in the methodological section, I recommend a brief presentation of methodological procedures in this work as well. I recommend presenting the statistical analysis separately.
Response to Reviewer #1 comments on Methods: Good idea. We have added a brief description of procedures as well as separating statistical analysis into a different section.

Reviewer #1 comment 1 on Discussion: p. 10, lines 334 – 341: The authors refer to their previous works, and therefore I recommend summarizing the results of the given works in the text or in the form of a Table.
Response to Reviewer #1 comment 1 on Discussion: You are right that results of our previous and current works need to be systematically reviewed and summarized in a more succinct and organized fashion. In this context, a review paper seems more appropriate. We feel that this goes beyond the scope of a research article. Here we focus on presenting new data and use our previous work mainly as a conceptual and methodological guide.

Reviewer #1 comment 2 on discussion: p. 12, section 3.9 *„Inferred pathogenic pathways...“:* I recommend including explanation of the pathogenic pathways also in the graphic form (as Figure).

<u>Response to Reviewer #1 comment 2 on Discussion</u>: We wish that we knew the pathogenic pathways leading the hypertension, with a possible exception of UMOD, uromodulin, that is involved in kidney phenotypes. As this point, pathways of the rest of the candidate genes are obscure. We just don't know. With caution, we merely quoting their possible functions from the literature, and refrained from making premature speculations on the pathways leading to hypertension at this writing. Our next stage of research is to prove by gene-targeting that each of these candidate genes is the QTL in question.

Comments of Reviewer #2:

Reviewer #2 general comments: The manuscript by Deng et al. "Shifting paradigm from gene expressions to pathways reveals physiological mechanisms in blood pressure control in causation" is very difficult to read and understand. I will demonstrate this problem by commenting the abstract, sentence by sentence.

Response to Reviewer #2 general comments: It seems that a comprehensive discussion is needed to clarify 'misunderstandings'. We divide general discussions into 3 sections.

First, the crux of our understandings relies on answering 2 questions: (1) "what does a QTL mean when not identified molecularly? (2) what is a QTL at the molecular level?" This issue has to be dealt with methodologically and mechanistically. Methods in detecting QTLs and mechanisms in regulating blood press are completely separate questions. Methods are tools that we use to detect QTLs, whereas mechanistically a QTL is equivalent to one gene when molecularly identified, or as you refer to as a quantitative trait gene (QTG).

Fundamentally at the molecular level, the principle of 1 QTL = 1 QTG = 1 gene stands. Please see 1.3 in Introduction for a succinct presentation. Our response to your comment 5, issue (3) later on will discuss this issue in depth.

Second, everything in our manuscript makes sense, if you'd use the molecular evidence supporting 1 QTL = 1 QTG = 1 gene, and evaluate our manuscript from that point of view. If you would insist on defining a QTL purely genetically, while ignoring its molecular nature, nothing would make sense. Do you have questions on the validity of any of our molecular data establishing 1 QTL = 1 QTG = 1 gene? We'd love to entertain any questions or objections you might have.

As elaborated in 3.2 of Discussion, there is a parallel between understanding QTLs in biology and understanding gravity in physics. If one views gravity from the Newtonian perspective of a force pulling on objects, the 'erratic' orbit of Mercury, origin of universe and black holes are nonsensical. However, if you view the same phenomena from the concept of curvature in warped space time of Einstein, they all fall into place (www.britannica.com/science/general-relativity).

Third, it appears that your evaluation of our manuscript started and ended at Abstract. The primary stumbling block seems to be what a QTL is. Our current understanding is that molecularly and mechanistically, the principle of 1 QTL = 1 QTG = 1 gene is valid.

We'd appreciate if you could evaluate our entire manuscript from Introduction, Results and Discussions. Most of your misunderstandings might have been alleviated. As you know in science, the most important aspect of research is to have the objective

and testable data. We'd appreciate it if you could evaluate, even challenge our data and interpretations of them presented in the manuscript.

Nevertheless, we are addressing every one of your specific comments as follows.

Reviewer #2 comment 1 on The abstract: "Genetic experimentations for blood pressure (BP) in human and animals have been partitioned into 2 separate specialties. However, this divide is mechanistically misleading."

Comments: It is not possible to perform genetic experimentation in humans, genetic analysis of blood pressure regulation in humans just uses different methods compared to animal models. Specifically, GWAS in humans provide sufficient statistical power to identify SNPs associated with blood pressure variability while studies in rodent models selected for susceptibility to high blood pressure use linkage analyses in genetically segregating populations to identify QTL which are confirmed in follow-up experiments in congenic strains.

Ideally, genes responsible for QTL are identified at the molecular level as variants of specific genes, so called QTG (quantitative trait genes). Both human and animal studies are usuful to understand the pathophysiology of hypertension. So it does not make sense that "…this divide is mechanistically misleading."

Response to Reviewer #2 comment 1 on the abstract: You are right that, methodologically, human GWAS and animal linkage studies use different genetic tools to localize chromosome regions probably containing QTLs of interest. Both human GWAS and animal linkage studies use powerful yet different statistical methods.

However, neither of the two types of differing methods deals with mechanistic questions of blood pressure regulation by a given QTL. As you have pointed out, this is the task of QTL identification by function at the molecular level that a gene/QTL has to be able to change blood pressure physiologically. Since little molecular identification of QTLs in humans has been achieved, we have to use examples from animal model studies. Limitations in human studies, as you stated, is that 'It is not possible to perform genetic experimentation in humans'.

As it turns out, mechanistically, a rodent QTL at the molecular level and its human molecular homologue, although initially detected by different methods, affect blood pressure by the same pathway. That is why differing methods in humans and animal studies can be mechanistically misleading. Namely, a mechanistic similarity could be shrouded and misinterpreted because of different methodologies used to find it.

A commonplace example of deceptive 'looks' that are mechanistically misleading is pencil lead and diamond. They look and feel differently. Pencil lead looks dark and can be easily peeled off, whereas diamond is shining and extremely hard. Yet,

fundamentally and mechanistically, they are both made of pure carbon, merely organized differently.

We can now return to QTL genetics and molecular identification of a QTL. In initial QTL mapping, linkage analysis in segregating populations tentatively found a probable rodent QTL in a broad segment containing multiple genes on rat Chromosome 17, designated as C17QTL (JCI. 93:2701). Follow up congenic knock in genetics separated the region containing C17QTL into 2 regions containing 2 separate QTLs, C17QTL1 and C17QTL2 (Hum Mol Genet. 22:4451). C17QTL1 was then found to belong to one of 4 genes (Hypertension 72:755).

By gene targeting, C17QTL1 was then molecularly identified to be a single gene, Chrm3 encoding [muscarinic cholinergic receptor 3 (M3R)] (Hypertension 72:755). No combination with another gene is necessary for C17QTL1/Chrm3 to affect blood pressure. Coincidentally by a different method, GWAS, a probable QTL was statistically marked by a SNP near CHRM3 in humans (Nature 447:661). As a result, different methods in 2 different orders of mammals converged to the same pathway of M3R signaling mechanistically regulating blood pressure (J. Hypertens. 38:322). Thus, the end point in QTL genetics by whatever methods used has to be a causative mechanism in physiologically modulating blood pressure. As exemplified by C17QTL1/Chrm3, 1 QTL = 1 QTG = 1 gene. The identity of the QTL leads to the mechanism.

Does this make sense?

Reviewer #2 comment 2 on The abstract: "BP physiology is mechanistically initiated by quantitative trait loci (QTLs)."

Comments: This sentence does not make sense. BP physiology cannot be mechanistically initiated by QTLs. The authors maybe wanted to say that identification of genetic determinants of blood pressure starts by mapping of QTLs to specific segments of chromosomes, followed by their genetic isolation in congenic strains and sublines.

Response to Reviewer #2 comment 2 on The abstract: The end point in QTL genetics by whatever methods used has to be a causative mechanism in physiologically modulating blood pressure. As exemplified by C17QTL1/Chrm3, 1 QTL = 1 QTG = 1 gene ((Hypertension 72:755). The identity of the QTL potentially leads to the physiological mechanism, e.g. M3R signaling. Thus, physiologically and in causation, 1 QTL/1 QTG/1 gene is a part of a pathway mechanistically controlling blood pressure. Does this make sense?

‘Initiated by’ may not be the right word. We have changed it to ‘participated by products of’. We hope the new sentence sounds more accurate: ‘BP physiology is mechanistically participated by products of quantitative trait loci (QTLs)’.

Reviewer #2 comment 3 on The abstract: “The key to unlock its mechanistic mystery lies in the past with mammalian ancestors before humans existed.“

Comments: This sentence does not make sense. Maybe the authors wanted to say that basic physiological mechanisms regulating blood pressure are evolutionary conserved between animal models and humans.

Response to Reviewer #2 comment 3 on The abstract: Your way of saying evolutionary conserved is correct, but avoids the origin of these conserved mechanisms. We’d like to ask you a deeper question: Where did basic physiological mechanisms regulating blood pressure come from that have become conserved during mammalian evolution?

This is an important question to address, because we know that a given mechanism of blood pressure control originated from common mammalian ancestors and became conserved during subsequent rodent and human evolution. In this sense, when we identify mechanisms regulating the current physiology in humans, we actually look into the mechanistic past before humans existed. We then need to integrate the evidence that rodents and humans have similar blood pressures, in spite of many physiological differences, such as size, life span etc. This means that, similar to those in rodents, physiological mechanisms controlling human blood pressure have already been given by ancestral QTLs in polygenic forms to virtually 100%.

When this fact is established, an inevitable conclusion will be that identifying rodent QTLs for blood pressure is equivalent to identifying the same human QTLs. We agree with your that analyzing QTLs in rodents is a lot more direct and powerful than analysing human QTLs due to experimental powers, such as gene targeting and designed inbreeding.

Specifically, the M3R signaling pathway in regulating BP existed in common ancestors of humans and rodents (Cardiol Cardiovasc Med 5: 471). Consequently, humans and rodents use the same pathway originating from their common ancestors and their similar BP states are not due to a convergent evolution event. This is because despite no M3R sequence is available from extinct common ancestors of humans and rodents 90 million years ago, the M3R signaling already existed in them (Cardiol Cardiovasc Med 5: 471).

3.6 of Discussion in the current manuscript elaborates this phenomenon further.

Does this make sense?

Reviewer #2 comment 4 on The abstract: "We hypothesize that humans and rodent share similar mechanisms."

Comments: Such hypothesis is useless. Many physiological and pharmacological studies have shown that basic physiological mechanisms regulating blood pressure are similar in animal models and humans.

Response to Reviewer #2 comment 4 on The abstract: Please see our response to your comment 3 above.

Reviewer #2 comment 5 on The abstract: "By shifting the focus from epidemiological after-effects to physiological causes, we have identified physiological mechanisms determining BP by QTLs."

Comments: I am not sure I understand the meaning of "epidemiological after-effects"; are they related somehow to GWAS? It is not possible to identify physiological mechanisms of blood pressure regulation by QTL mapping. QTL is a segment chromosome usually with multiple genes. Identification of a QTL provides information about location of putative genetic determinants (not specific genes) regulating blood pressure but provides no information about physiological mechanisms regulating blood pressure. Maybe authors wanted to say that physiological analysis of congenic strains with genetically isolated QTL might provide some hints about physiological mechanims related to blood pressure diffrence.

Response to Reviewer #2 comment 5 on The abstract: Your comments deal with 3 issues, (1) causes vs after effects; (2) identifying a physiological mechanism of a QTL; (3) molecular definition of a QTL. We'll address each of these 3 issues as follows:

(1) For a discussion comparing causes and after effects, 3.2 of Discussion elaborates this aspect in detail.

(2) You are right to point out limitations of pure QTL mapping without follow up molecular studies to uncover physiological mechanisms of blood pressure control. QTL mapping serves only as an entry point for further functional analyses including congenic knock ins and gene targeting. Identifying the M3R signaling pathway via studying C17QTL1 is a prime example. From that, a physiological mechanism by a QTL is indeed identified as detailed to you in our response to your comment 3 above.

(3) 1.3. of Introduction defines a QTL genetically and molecularly. Here we elaborate it by using C17QTL1/Chrm3 as a case in point.

Via QTL mapping, a QTL can only be vaguely defined as a locus present in a segment of chromosome. For instance, linkage analysis in segregating populations

tentatively found a probable rodent QTL in a broad segment containing multiple genes on rat Chromosome 17, designated as C17QTL (JCI. 93:2701). Follow up congenic knock in genetics separated the region containing C17QTL into 2 regions containing 2 separate QTLs, C17QTL1 and C17QTL2 (Hum Mol Genet. 22:4451). C17QTL1 was then found to belong to one of 4 genes (Hypertension 72:755).

By gene targeting, C17QTL1 was then molecularly identified to be a single gene, Chrm3 (Hypertension 72:755). No combination with another gene is necessary for C17QTL1/Chrm3 to affect blood pressure. Thus, the end point in QTL genetics has to be molecularly identifying a QTL and its causative mechanism in physiologically modulating blood pressure. As exemplified by C17QTL1/Chrm3, 1 QTL = 1 QTG = 1 gene. Do you have any objections to the molecular evidence supporting this conclusion? It's all in the literature.

Without sounding too preachy, in science, it is essential to follow the updated evidence already existing in the literature.

Therefore, your ***genetic*** sense of a QTL as a segment of a chromosome containing many genes was more or less passable for initial QTL mapping 15-30 years ago. During that time, few QTLs were molecularly known. Here we focus on QTLs for blood pressure. It was evident from years of 1990s to 2000s (Science 265:2037; Hypertension 25:1121; Physiol. Rev. 80:135).

Since then, a number of QTLs for blood pressure have been ***molecularly*** identified in animal models. The true meaning of a QTL of physiological importance has emerged and been updated to our molecular understanding, namely, 1 QTL = 1 QTG = 1 gene. In this manuscript, we define 1 QTL = 1 QTG = 1 gene according to our current insights, despite the fact that most QTLs have not been molecularly identified.

One may wonder why don't we rename a QTL to a QTG when molecularly identified? How about the name of the gene responsible for the QTL/QTG? It opens a can of semantic 'worms', rather than being substantive.

Our updated understanding of a QTL from the genetic to molecular insight mirrors our progressive understanding of a gene. Before any gene was molecularly identified, the term was coined by Johannsen to describe the Mendelian units of heredity. Now we understand that a gene is a stretch of DNA molecules encoding a protein product. In this chemical sense, we continue to use the term gene to describe DNA sequences as a Mendelian factor or a QTL for blood pressure.

Although there is nothing wrong calling a gene purely as a Mendelian factor, it is grossly inadequate, because our current molecular understanding of it exceeds it and

delves deeply into mechanisms. Namely, a gene codes for a protein product that can participate in a pathway affecting a phenotype of interest, e.g. blood pressure. The most important scientific research of a gene, or a QTL for that matter, is to unravel its molecular and physiological function.

The understanding of a QTL requires us to identify its molecular mechanism that goes beyond a vague scope of QTL mapping to a segment of a chromosome. Based on our molecular understanding of a QTL, even in QTL mapping, the most appropriate description should be that a QTL is localized to reside in a segment of a chromosome. Before molecular identification, a QTL is a locus in a chromosome segment, not equal to a chromosome segment. It's like using street signs to locate a house. Streets are not the same as the house itself.

There are many examples in science that the outdated knowledge is no long valid in the present context of our understandings. First, before 1950, the molecular nature of a gene was believed to be protein. Since then, we know that DNA is the fundamental chemical makeup of a gene. If one continues to treat a gene from the outdated view of protein, it is incorrect. Second, if one uses an almanac dated 2000 to call Pluto a planet, it's no longer valid because of what we know today. Third, if someone uses a text book dated 1910 which described gravity as a force, we know today gravity is not a force, but a curvature in space time. The list goes on and on.

In a mundane analogy, one can compare finding a QTL to diamond mining. We know what diamonds are as we define them chemically, although they have not been unearthed yet. We don't think of diamond as something else, when it's mixed with other stuff in the ground. An area containing a diamond (analogous to a segment of a chromosome containing a QTL) is not diamond (QTL) itself. The fact of not finding a diamond does not repudiate what a diamond is.

Likewise, 1 QTL = 1 QTG = 1 gene is what a QTL is in chemistry. Whether or not a QTL is molecularly identified does not nullify the molecular nature of it. Our recent understanding of 1 QTL = 1 QTG = 1 gene is fundamental in QTL research, because it leads to mechanistic insights.

At present, many QTLs determining polygenic, quantitative, complex and common traits have not been molecularly identified. This lack of information can not, and does not, refute what a QTL is chemically, i.e. 1 QTL = 1 QTG = 1 gene. We are respectfully requesting that you evaluate our manuscript based on this state-of-the-art principle of a QTL. Everything will make sense from this perspective.

Primarily in response to your comments, a brief version of the above discussion has been added to 1.3 of Introduction.

More evidence on 1 QTL = 1 QTG = 1 gene is presented in our response to your comment 6 next.

Reviewer #2 comment 6 on The abstract: "The evidence has reproduced the outcome that each QTL genetically acts as a building block by Mendelian monogenicity."

Comments: This phrase that "…QTL genetically acts…by Mendelian monogenicity" is an oxymoron.

Response to Reviewer #2 comment 6 on The abstract: In fact, we can prove it to you that 1 QTL = 1QTG = 1 gene acts as a truly Mendelian factor as if a QTL determines a monogenic phenotype. Multiple QTLs are organized in modules/groups to achieve polygenicity of blood pressure. 5. Conclusion in the manuscript has more.

To understand this further, we'd like you to treat a QTL/QTG/gene as a Mendelian 'carbon' element. Multiple QTLs/'carbon' elements are organized to form different poly-carbon materials, such as graphites (one polygenic trait) and diamonds (another polygenic trait). In all these different polyforms, the basic building block is the same, a Mendelian monogenicity/ 'carbon'.

This is a ground-breaking discovery!!! (nothing contradictory at all).

Now, we'd like to present definitive proofs, as long as you do not object to the recent molecular meaning of 1 QTL = 1 QTG = 1 gene [see our response to your comment 5, issue (3) above]. We'd respectfully request that you would critically examine the molecular evidence supporting this meaning in the literature. We'd be happy to respond to any questions and challenges that you may have.

In the following, we present some, although not exhaustive, proofs for the principle of 1 QTL = 1QTG = 1 gene and each of them acts as if it controls a Mendelian monogenic trait. Each of these QTLs has been definitively proven to change blood pressure by gene targeting and published in the literature. Genes for other QTLs that have not been proven by gene targeting are not included here.

Proof #1 for 1 QTL = 1 QTG = 1 gene: C17QTL1/Chrm3 [Please see our response to your comment 5, issue (3) above]. Further, blood pressure of heterozygous Chrm3+/- rats is the same as blood pressure of Chrm3+/+ homozygotes (Hypertension 72:755). This heterozygote dominance is typical of a Mendelian character.

Proof #2 for 1 QTL = 1 QTG = 1 gene: C10QTL2 is encoded by the gene of Rffl (E3 ubiquitin-protein ligase rififylin) (PLoS Genet 13:e1006961).

Proof #3 for 1 QTL = 1 QTG = 1 gene: C1QTL1 is encoded by the gene of Adamts16 (A disintegrin-like metalloproteinase with thrombospondin motifs–16) (PNAS 109: 20555).

In conclusion, there is no doubt that the principle of 1 QTL = 1 QTG = 1 gene is valid, much the same way as a gene is made up DNA molecules. Based on this principle, we can deepen our understandings of the molecular nature for each of blood pressure QTLs that we have analyzed so far (2.4 of Results and 3.4 of Discussion).

It is in the same fashion that we understand what a gene is in chemistry. Since the molecular identity of a gene as a track of DNA sequence is established to encode a protein that may play a role in physiological functions, we understand how a gene determines Mendelian inheritance more profoundly and mechanistically than a mere an abstract idea of a gene.

Reviewer #2 comment 7 on The abstract: "A gene dose for a QTL is irrelevant to BP controls."

Comments: A QTL is not a gene, thus "a gene dose for a QTL…" does not make sense

Response to Reviewer #2 comment 7 on The abstract: Please see our response to your comment 5, issue (3) above. 1 QTL = 1 QTG = 1 gene! Please look at the evidence presented in our response to your comment 6 above. Does it make sense?

Reviewer #2 comment 8 on The abstract: "Together, QTLs join one another as a group in modularized Mendelian fashion to achieve polygenicity."

Comments: According to the authors a group of QTLs is similar to a Mendelian trait and thus achieves polygenicity. It does not make sense.

Response to Reviewer #2 comment 8 on The abstract: Please see our response to your comment 6 above.

Reviewer #2 comment 9 on The abstract: "Mechanistically, the QTLs in the same module appear to function in a common pathway. Each is involved in a different step in the pathway towards polygenic hypertension."

Comments: QTLs are segments of chromosomes with many genes. Thus it is not possible to analyze mechanisms of chromosome segments.

Response to Reviewer #2 comment 9 on The abstract: Please see our responses to your comments 5 issue (3) and 6 above. 1 QTL = 1 QTG = 1 gene!

Mechanistically, the M3R signaling pathway has been shown to play an important role in blood pressure controls, because M3R is encoded by C17QTL1/Chrm3 alone.

Reviewer #2 comment 10 on The abstract: "This emerging concept is a departure from the human-centric precept that the level of QTL expressions, not physiology, would ultimately determine BP."

Comments: According to the authors, GWAS are human-centric because they use common SNPs that are specific to humans and are not present in rodent models. The authors argue that these polymorphisms cannot represent genetic variants regulating blood pressure. However, this is misunderstanding, nobody claims that common variants used in GWAS are reponsible genes. And of course, GWAS are human-centric.

QTLs are segments of chromosomes and therefore "QTL expressions" is a nonsense.

Response to Reviewer #2 comment 10 on The abstract: Once again, please see our responses to your comments 5 issue (3) and 6 above. 1 QTL = 1 QTG = 1 gene! Please look at the evidence. Does the evidence make sense?

We'd like to bring your attention to the current status on human GWAS (Boyle et al. An Expanded View of Complex Traits: From Polygenic to Omnigenic. *Cell* 169, 1177). In their view, GWAS SNPs would have been practically responsible for QTLs for the phenotype in question, e.g. blood pressure. Each of them would have a miniscule effect on the phenotype. Together via gene expressions, they would determine the phenotype. As you know, there is no functional proof that any of these GWAS SNPs could physiologically affect the phenotype in question, e.g. blood pressure in causation.
